# The Efficacy of Group Metacognitive Therapy on Self-Esteem and Mental Health of Patients Suffering from Major Depressive Disorder

**Published:** 2014

**Authors:** Vahid Farahmand, Ramezan Hassanzadeh, Bahram Mirzaian, Mohammad Reza Fayyazi Bordbar, Jaleh Feizi

**Affiliations:** 1 Department of Psychology, School of Humanities, Sari Branch, Islamic Azad University, Sari, Mazandaran, Iran.; 2 Associate Professor, Psychiatry and Behavioral Sciences Research Center, Mashhad University of Medical Sciences, Mashhad, Iran.; 3 Department of Psychology, School of Medicine, Mashhad University of Medical Sciences, Mashhad, Iran.

**Keywords:** Major Depressive Disorder, Mental Health, Metacognitive Therapy, Self-esteem

## Abstract

**Objective:** The present research aims to analyze the efficacy of group metacognitive therapy (MCT) on self-esteem and mental Health of those who suffer from major depressive disorder.

**Methods:** The research was a randomized clinical controlled trial, using pretest and posttest with 2 months of follow-up. Twenty-two patients with major depressive disorder based on DSM-IV-TR criteria were selected through available sampling from patients of two psychiatric hospitals of Mashhad, Iran, in 2011. They were allocated randomly into two groups of trial (n = 11) and control (n = 11). Citalopram and sertraline were prescribed as antidepressant to both groups. The experimental group also attended nine 90-minute sessions of MCT (a 5-week program). Eysenck self-esteem scale (ESES) and Mental Health Checklist (MHC) were used in pretest, posttest, and follow-up as the study instrument. The data were analyzed by analysis of covariance (ANCOVA) using SPSS.

**Results: **ANCOVA revealed that the patients receiving group MCT had significantly increased (p < 0.001) self-esteem in posttest, which remained significant in the follow up (p < 0.001). Moreover, mental health scores had increased in the MCT group (posttest, p < 0.001; follow up, p < 0.001).

**Conclusion:** Group MCT has beneficial therapeutic roles in improving the self-steam and recuperation of mental health in MDD patients.

## Introduction

Negative biases in data processing about self have been known as one of the major characteristics in developing and maintaining of long-term clinical depression (1). Depressed patients encounter a situation in which they are not assertive enough to move, but they complain and are disappointed with the situation they were in (2). Because of the recurrent and chronic course of depression, and the potential disability caused by it, knowing the risk factors and taking immediate action in early stages are of paramount importance (3).

Self-esteem is one of the most important aspects of the personality and determines the human behavior specifications (4). Deb and Bhattacharjee state that depressed people’s self-esteem is significantly lower than ordinary people (5). People with lower self-esteem are more vulnerable to depression; thus, depression can be considered as the result of low self-esteem (6).

Conditions of psychological disorders and their prevalence in adulthood have presented distressing figures. For instance according to Bahrami, Beck has reported that depression has drastically spread during the recent decades and has become a detrimental issue (7). Moreover, the International Health and Hygiene Organization has estimated that depression and anxiety disorder will be at the top of the list of psychological disorders and will include 25% of the patients going to the health care centers in the world (8).

The remarkable subject is the interest of therapists in giving meaning to the cognitive phenomena in patients suffering from anxiety and depression. Metacognition is the knowing cognition of the cognition (1). The metacognitive approach has been created because of dissatisfaction with the existing approaches to psychotherapy which do not consider the important inadaptable thinking specifications and their controlling factors (9). This approach focuses on mental processes of thinking style (10). Wells and Matthews have introduced the Self-Regulatory Function model (S-REF) to determine the psychological disorders as a fundamental model of treating the metacognition (11). Considering this model, conserving the disorder is related to activating a specific style of thinking called Cognition Attentional Syndrome (CAS) (9). The important symptom of this syndrome is the constancy of negative thoughts in a form of rumination or worry (12). This mental pattern has a crucial role in triggering emotional disorders, especially depression, and also in recognizing its causes (13). From this point of view, these challenging procedures have reverse effects on emotional disorders (12). This syndrome has various consequences, which lead to psychological disorders. The psychologist must carefully diagnose CAS and name it for the patient’s awareness and knowledge during the treatment session (13). 

Alloy et al. considered mental rumination as a description and interpretation of stressful incidents, and introduced it as mental rumination to react against stress (14). 

This style of thinking often occurs in response to fundamental automatic negative thoughts. The difficulty of this rumination is that it has long term negative and stressful effects, and the individual’s concentration will be on ideas and processes, which reinforce ineffective knowledge (10). This kind of thinking (rumination) causes pessimistic interpretations of life events, leading the individual to be also pessimistic about future positive events and making them weak so that he/she will be unable to solve interpersonal problems (12). It also causes an increase in the negative mood effects on motivation and problem solving (for example through self-doubt regarding the ability to solve the problem or the cognitive abilities) (15).

One presumption is that instead of changing the cognitive content that is used in cognitive approach, we must decrease its useless effects by trying to change the styles used for experiencing the mental events (16).

MCT enables the patients to stop rumination, decrease self-monitoring, and create more adaptable styles to encounter their thoughts and emotions. The attention training technique (ATT) is one of the techniques used that do not involve shifting attention to neutral or positive stimuli to control or avoid subjective experiences. Instead ATT involves shifting attention in ways that are specifically designed to strengthen metacognitions that regulate thinking, remove unhelpful thinking styles that impede normal emotional processing, or modify beliefs (16). The important part of the treatment is changing the negative and positive metacognitive beliefs about rumination, and changing or eliminating Cognitive Attentional Syndrome (CAS) (13). Positive metacognitive beliefs are concerned with the usefulness of worrying, rumination, threat monitoring, and other similar strategies. The second domain of metacognitive beliefs is concerned with the negative significance and meaning of internal cognitive events, such as thoughts and ordinary beliefs (15).

Previous researches have shown that there is an association between high self-esteem and positive character specifications in individuals. People with high self-esteem have specifications such as mental maturity, stability, being realistic, tranquility, and high capability of tolerating failure and disappointment. However, people with low self-esteem do not display such specifications. As was mentioned, low self-esteem is remarkably connected to pathological symptoms and has an association with many of the mental health issues and problems and usually leads to mental traumas (17). Therefore, by considering the importance of self-esteem and its key role in mental hygiene, and also the need for designing innovative treatments, we decided to perform the present study with the purpose of assessing the efficiency of group MCT on self-esteem and mental health of the people with major depression.

## Materials and Methods

The present study is an open labeled, randomized control trial conducted in Mashhad, the second biggest city of Iran. In this study the pretest–posttest and follow-up plan with the control group has been used. The study population included the patients diagnosed with major depressive disorder (MDD), based on DSM-IV-TR criteria, who were referred to Ghaem and Ibn-e-Sina psychiatric hospitals of Mashhad in 2011 with the age of 18 to 50 years. 

30 patients have been chosen by available (convenient) sampling. A clinical psychologist and psychiatrist approved their diagnostic of MDD based on DSM-IV-TR criteria. 

Inclusion and exclusion criteria are: 1- meeting DSM-IV-TR criteria of MDD based on semi-structured interview, 2- lack of borderline personality disorder, 3- not receiving any other psychological intervention, 4- not having any other psychological therapy at least during the 2 previous years, 5- no history of psychosis, 6- no substance abuse or dependency, and 7- being between 18-50 years of age. Patients were allocated randomly by using table of random numbers into 2 groups; trial group (n = 15) and control group (n = 15). The participants started pharmacotherapy by SSRIs (citalopram or sertraline). Citalopram was started with the initial dose of 10 mg/d, and increased 10 mg every 3 days up to 40 mg/d. Sertraline was started with the initial dose of 50 mg/d, and increased 50 mg every 3 days up to 150 mg/d. Four weeks after the beginning of drug treatment, the experimental group started the MCT sessions. The sessions were conducted by two clinical psychologists (a therapist and a co-therapist). In the second treatment session, we encountered the drop out of 4 patients (3 males and 1 female). Their reasons were the dissatisfaction of their wife or being busy for final exams. Thus, 4 patients from the control group (4 females) were randomly omitted.

Eysenck self-esteem scale (ESES) and Mental Health Checklist (MHC) were used as measures for assessing the outcomes. These tools were used in pretest (before starting the group metacognitive therapy and 4 weeks drug therapy), posttest (end of sessions), and 2 months of follow-up. We have to mention that during the first interview the essential information of to the research was given to the patients. In addition, they were convinced that the data would be published for research purposes only. Then, the informed consent form was handed over to them, in which some notes such as treatment description, negative and positive effects, supplementary treatments, and inclination to leave the treatment plan in any desired period of time were included. Moreover, it was prescribed that the control group also take the course of the MCT group, after the research is over. 

The sessions’ contents are presented in the table 1. We have to mention that as the first session was one of the most important treatment sessions, to promote the possibility and quality of properly formulizing the participants, the therapist felt the necessity to hold this session individually for each of the members of the group. During all the sessions, if necessary the patients were in touch with the therapist. Furthermore, the therapist was in touch with each participant during the interval of sessions to make sure they performed their personal assignments with a favorable quality. Ultimately, the research data were analyzed by modifying statistical indices and analysis of covariance (ANCOVA), using SPSS for Windows 16.0 (SPSS Inc., Chicago, IL., USA). Data are expressed as means (± SD) and p < 0.05 is considered statistically significant. The study was approved by the Medical Research Ethics Committee of Islamic Azad University of Sari.

**Table 1:** The content of MCT sessions on self-esteem and mental health of the patients suffering from major depression

**Sessions**	**Session’s content**
**1**	Formulizing and practicing the attention training techniques (ATT). This session was held individually.
**2**	Reviewing the individual assignments done during the previous session, careful identification rumination, practicing detached mindfulness (DM), challenging negative MC beliefs about rumination, practicing ATT, and determining the next session’s individual assignments.
**3**	Reviewing the previous session’s individual assignments, continuing the challenge of negative MC beliefs and practicing ATT, and determining the next session’s Individual assignments.
**4**	Reviewing the previous session’s individual assignments, continuing the challenge of negative MC beliefs related to the rumination being uncontrollable and practicing ATT, and determining the next session’s individual assignments.
**5**	Reviewing the previous session’s individual assignments, challenging the positive MC beliefs and practicing ATT, and determining the next session’s individual assignments.
**6**	Reviewing the previous session’s individual assignments, continuing the positive challenge of the MC beliefs and practicing ATT, and determining the next session’s individual assignments.
**7**	Reviewing the previous session’s individual assignments, studying and changing incompatible behavior, practicing ATT, and determining the next session’s individual assignments.
**8**	Reviewing the previous session’s individual assignments, challenging negative beliefs about depression symptoms, practicing ATT, and determining the next session’s individual assignments.
**9**	Reviewing the previous session’s individual assignments, collecting a new program to prevent the outbreak, scheduling the follow-up session, and practicing ATT.

***Research tools***

*Eysenck Self-esteem Scale*

This scale includes thirty questions of two characteristic types related to self-esteem, compatible stable type and emotionally instable type. Compatible stable type has been formed by attributes such as self-esteem, happiness, tranquility, and etcetera. The emotionally instability type has been formed by attributes such as inferiority feeling, depression, anxiety, and etcetera. The subjects must try to answer yes or no as much as possible for each question; if it is not possible for them, they must choose the third choice and choose the question mark. In this scale the highest mark for the testing is 30; the higher scores show high self-esteem (18). Cronbach’s Alpha coefficient in this research was 0.83. 

*Mental Health Checklist (MHC)*

This checklist has been provided by Kumar in order to provide a hand tool to identify people who have poor mental health. The reliability coefficient of this test was 0.83 (19). The checklist evaluates six mental conditions and five physical conditions through Likert scale. Score ‘A’ stands for mental condition score, and score ‘B’ pertains to physical condition score, and by adding these two scores a total score will be obtained. High scores show poor mental health (20). Cronbach’s alpha coefficient for the whole questionnaire in this research was 0.72.

## Results

The participants in this research were in the age range of 18 to 50 years. The mean (± SD) age of the trial group was 30.36 (± 10.39) and the control group was 31.73(± 9.61) (p > 0.05). There were 1 male and 10 female participated in the trial group, and 1 male and 10 female participated in the control group. Both groups were using drugs (with the mean dose of 110 mg/d of sertraline in 5 patients and mean dose of 40 mg/d of citalopram in 6 patients) at the same time.

Table 2 shows the descriptive data of the variables of self-esteem and mental health which are separated for groups and stages.

**Table 2:** Descriptive indexes of self-esteem and mental hygiene in pretest, posttest, and the follow-up stage. Data are displayed as means (± SD

	**Trial Group**			**Control Group**		
**Variables**	**Pretest**	**Posttest**	**Follow up**	**Pretest**	**Posttest**	**Follow-up**
**Self-esteem**	Mean (± S.D)	Mean (± S.D)	Mean (± S.D)	Mean (± S.D)	Mean (± S.D)	Mean (± S.D)
**Mental Health**	12.31 (± 6.25)	23.77 (± 3.93)	24.72 (± 1.73)	12. 04 (± 2.94)	16.50 (± 2.81)	19.31 (± 3.57)
**Self-esteem**	31.00 (± 3.79)	18.09 (± 4.01)	15.09 (± 3.50)	33.91 (± 5.37)	27.45 (± 4.61)	30.36 (± 10.39)

**Table 3:** Summary of ANCOVA for the treatment effect on self-esteem and mental hygiene through controlling the pre-test scores [After controlling the pretest scores, there was significant differences between two interfering groups in the post-intervention scores (posttest and follow-up)].

**Variable**	**Stages**	**Source**	**df**	**Mean Square**	**F**	**Sig.**	**Partial Eta Squared**
**Self-esteem**	Post-test	Pretest	1	062.91	06.99	0.010	0.26
		Group	1	282.80	31.46	0.000	0.62
	Follow-up	Pretest	1	017.17	02.31	0.140	0.10
		Group	1	157.72	21.23	0.000	0.52
**Mental Health**	Post-test	Pretest	1	119.50	08.93	0.008	0.32
		Group	1	304.86	22.79	0.000	0.54
	Follow-up	Pretest	1	018.63	01.86	0.180	0.89
Group	1	413.38	41.35	0.000	0.68

Table 3 shows the results of ANCOVA, to compare the posttest scores and follow-up data of the two groups after controlling for the pretest. The analysis of covariance has been planned to assess the effectiveness of the MCT, which was implemented for the improvement of self-esteem and mental health. In the implementation of the pre-intervention self-esteem and mental health test, the patients’ scores were used as covariates in this analysis.

The preliminary studies were done to make sure of not offending against normal distribution, homogeneity of variance, homogeneity of regression slopes, and the constancy of the covariate variant measurements. After balancing the pre-intervention (pretest) scores, there was a significant difference between the two intervention groups in the post-intervention scores (posttest and follow-up) of the self-esteem scale (posttest: Partial Eta = 0.62; p < 0.001; F (1, 19) = 31.46, and follow-up: Partial Eta = 0.52; p < 0.001; F (1, 19) = 21.23). These results are also true for the mental health test (follow-up: Partial Eta = 0.68; p < 0.001; F (1, 19) = 41.35, and posttest: Partial Eta = 0.54; p < 0.001; F (1, 19) = 22.79).

## Discussion

The patients undergoing MCT intervention showed significantly higher rates of recovery in comparison to the control group. Moreover, in the follow-up period the results remained significant. No studies regarding assessment of the MCT effectiveness in improving self-esteem and mental health were found in our searches however, the most related researches are explained below.

Wells et al., in their research, studied MCT effectiveness on depressed patients (n = 4) through 3 and 6 months of follow-up (9). The findings of this research showed a significant decrease in depression symptoms and also continuation of the treatment in the next 3 and 6 months follow-up. In the studies by Wells et al., through using basic multi-line A-B plan, they studied the MCT effectiveness on depressed patients. The treatment was given to the patients during 6–8 sessions. A significant recovery was observed in the patients; the mean score of BDI-II of the pre-test was 23.35, but reached 6.5 after treatment. In addition, in the six months follow-up all the patients fulfilled the standard recovery criteria of the Beck depression scale (10). Furthermore, Wells et al. found that under the MCT intervention 66% of patients suffering from major depressive disorder had completely recovered during the six months follow-up (9). In another research, which used ATT as the drug supplementary treatment for the depressed patients (n = 15), results showed that this treatment was better than ordinary treatment (anti-depression drugs) (21). 

In line with the current research, Dowlatshahi performed a research on the effectiveness of MTC in depressed patients (n = 10). The treatment was given during 8 sessions and led to a considerable recovery of patients; the mean percentage of recovery from depression symptoms was 35% in the trial group. In addition, ANCOVA results in this study were significant (p < 0.001) (22). Hashemi et al. studied the efficiency of MCT on the recovery of patients with major depressive disorder (n = 3) during 6–8 sessions with 1 and 3 months follow-up. Their findings indicate significant and considerable changes in all three patients’ depression symptoms; the mean score of the BDI-II decreased from 23.1 before the treatment to 6.3, 6, and 12.4 immediately after the treatment, and during the one month and three month follow-up, respectively (23).

Discrepancies between the results of the current study and other studies in symptom responses to MCT may be due to methodological reasons, various outcome measures, and cultural differences. 

The results obtained in this research were encouraging; suggesting MCT as a short-term treatment of severe depression either independently or in combination with pharmacotherapy. We must mention that none of the patients reported that the symptoms became worse during the treatment course; except for one person, who reported that the symptoms worsened during the 3^rd^ and 4^th^ sessions, but this person recovered remarkably at the end of the treatment and maintained this state during the follow-up. In conclusion, we can consider MCT as an effective method because of paying attention to some factors in controlling and experiencing negative thoughts such as: why people produce negative thoughts and extend them, focusing on the mental processes of the thinking style, the metacognitions contents not to the negative automatic thoughts contents and schemas, and focusing on the procedural change (10, 24).

This research had some limitations which can be mentioned. Because one of the participants did not attend 2 group sessions, these 2 sessions were held individually for him/her. The small sample size (n = 22), the age range of 18-50 years that can limit the generalization of the research findings, short-term follow-up (2 months), and the limited numbers of researches in this field for better comparisons are the other limitations we encountered.

## Conclusion

This study, for the first time, showed that depressed patients receiving MCT showed significantly increased self-esteem and mental health in posttest after controlling the pre-test scores, which is in agreement with previous studies supporting the role of MTC in treatment of patients.

## Footenote

**Declaration of interest:** None.

**Clinical Trial Registration-URL: **http://www.irct.ir. Unique identifier: TRCT201201162611N3.
